# Motor Skill Acquisition Promotes Human Brain Myelin Plasticity

**DOI:** 10.1155/2016/7526135

**Published:** 2016-05-16

**Authors:** Bimal Lakhani, Michael R. Borich, Jacob N. Jackson, Katie P. Wadden, Sue Peters, Anica Villamayor, Alex L. MacKay, Irene M. Vavasour, Alexander Rauscher, Lara A. Boyd

**Affiliations:** ^1^Department of Physical Therapy, University of British Columbia, Vancouver, BC, Canada V6T 1Z3; ^2^Division of Physical Therapy, Department of Rehabilitation Science, School of Medicine, Emory University, Atlanta, GA 30322, USA; ^3^Department of Physics & Astronomy, University of British Columbia, Vancouver, BC, Canada V6T 1Z3; ^4^Graduate Program in Rehabilitation Sciences, University of British Columbia, Vancouver, BC, Canada V6T 1Z3; ^5^Department of Radiology, University of British Columbia, Vancouver, BC, Canada V6T 1Z3; ^6^Department of Pediatrics, University of British Columbia, Vancouver, BC, Canada V6T 1Z3; ^7^Child & Family Research Institute, University of British Columbia, Vancouver, BC, Canada V6T 1Z3; ^8^Centre for Brain Health, University of British Columbia, Vancouver, BC, Canada V6T 1Z3

## Abstract

Experience-dependent structural changes are widely evident in gray matter. Using diffusion weighted imaging (DWI), the neuroplastic effect of motor training on white matter in the brain has been demonstrated. However, in humans it is not known whether specific features of white matter relate to motor skill acquisition or if these structural changes are associated to functional network connectivity. Myelin can be objectively quantified* in vivo* and used to index specific experience-dependent change. In the current study, seventeen healthy young adults completed ten sessions of visuomotor skill training (10,000 total movements) using the right arm. Multicomponent relaxation imaging was performed before and after training. Significant increases in myelin water fraction, a quantitative measure of myelin, were observed in task dependent brain regions (left intraparietal sulcus [IPS] and left parieto-occipital sulcus). In addition, the rate of motor skill acquisition and overall change in myelin water fraction in the left IPS were negatively related, suggesting that a slower rate of learning resulted in greater neuroplastic change. This study provides the first evidence for experience-dependent changes in myelin that are associated with changes in skilled movements in healthy young adults.

## 1. Introduction

Until recently, investigations of motor skill acquisition and neuroplastic change focused primarily on cortical grey matter [1]. Notably, there is an absence of research investigating how underlying components of white matter (e.g., myelin) interact with neurophysiology or functional connectivity to support motor skill acquisition. Previous work exploring experience-dependent changes in white matter relied on diffusion weighted imaging (DWI) [2, 3] or rodent models [4, 5]. Though data from past work in humans are useful in grossly characterizing diffusion behavior influenced by microstructural components of white matter, multiple structural features can be individually or collectively responsible for the observed changes in DWI measures, including (1) axonal membrane status, (2) myelin sheath thickness, (3) number of intracellular neurofilaments and microtubules, and (4) axonal packing density [6]. Thus, while DWI is useful in grossly assessing white matter microstructure, it has limited utility in localizing changes in specific neurobiological components.

Myelin is crucial for rapid neuronal signal conduction and modifiable by experience [7]. In addition, motor skill dysfunction is a cardinal symptom of myelin related diseases, such as multiple sclerosis [8]. Recent work demonstrated that oligodendrocyte precursor cell proliferation and myelin structure are associated with behavioural changes in rodent models [9]. Yet relatively little is known about experience-dependent changes in myelin, specifically whether skilled motor training stimulates plasticity in brain myelin in humans. This lack of knowledge stems in part from technological limitations that until recently prevented the imaging of myelin* in vivo*. However, the recent development of new noninvasive imaging techniques can quantitatively characterize changes in myelin in the human brain [10]. Noninvasive,* in vivo* characterization of human brain myelin is possible using multicomponent T2 relaxation imaging (MCRI). MCRI separates the magnetic resonance proton signal relaxation time into three components: (1) a long T2 component (>2 s) that is attributed to cerebral spinal fluid, (2) an intermediate T2 component (~80 ms) attributed to intra/extracellular water, and (3) a short T2 component (<40 ms) that reflects water trapped between myelin lipid bilayers (myelin water fraction, MWF) [10, 11]. Formalin-fixed human brains yield T2 distributions similar to those found* in vivo*, and histopathological studies show strong correlations between MWF and staining for myelin [12, 13]. To date, no work has examined the relationship between myelin plasticity and motor skill acquisition. Therefore, the objective of the current study was to characterize experience-dependent changes in myelin following visuomotor skill training in healthy human volunteers.

## 2. Materials and Methods

Seventeen right-hand dominant, healthy young adults (seven males; mean age 26 ± 4 years), with no history of musculoskeletal or neurological injury that would affect task performance, provided informed consent in accordance with the Declaration of Helsinki. The University of British Columbia research ethics boards approved all aspects of the study protocol. Handedness was assessed by asking participants which hand they used to write with most frequently. Participants completed a baseline MRI acquisition session no more than 24 hours prior to the first of 10 separate motor skill-training sessions and then a follow-up MRI acquisition session within 24 hours of the last training session, over the course of four weeks.

### 2.1. MRI Acquisition

Magnetic resonance data were acquired at the University of British Columbia MRI Research Centre and were obtained on a Philips Achieva 3.0 T whole body MRI scanner (Phillips Healthcare, Best, NL) using an eight-channel sensitivity encoding head coil and parallel imaging. The following scans were collected: (1) 3D T1 turbo field echo (TFE) scan (TR = 7.4 ms, TE = 3.7 ms, flip angle *θ* = 6°, FOV = 256 × 256 mm, 160 slices, 1 mm slice thickness, scan time = 6.0 min), (2) 60-orientation diffusion weighted scan with a single shot echo-planar imaging (EPI) sequence (TR = 7013 ms, TE = 60 ms, FOV = 224 × 224 mm, 70 slices, 2.2 mm slice thickness, voxel dimension = 2.2 mm^3^, *b* = 700 s/mm^2^, and scan time = 7.4 min) and 5 *b* = 0 s/mm^2^ images, and (3) whole-cerebrum 32-echo three-dimensional gradient- and spin-echo (3D GRASE) for T2 measurement (TR = 1000 ms, echo times = 10,20,30,…, 320 ms, 20 slices acquired at 5 mm slice thickness, 40 slices reconstructed at 2.5 mm slice thickness (i.e., zero filled interpolation), slice oversampling factor = 1.3 (i.e., 26 slices were actually acquired but only the central 20 were reconstructed), in-plane voxel size = 1 × 1 mm, SENSE = 2, 232 × 192 matrix, receiver bandwidth = 188 kHz, axial orientation, and acquisition time = 14.4 min) [10].

### 2.2. Motor Task

**Figure 1:**
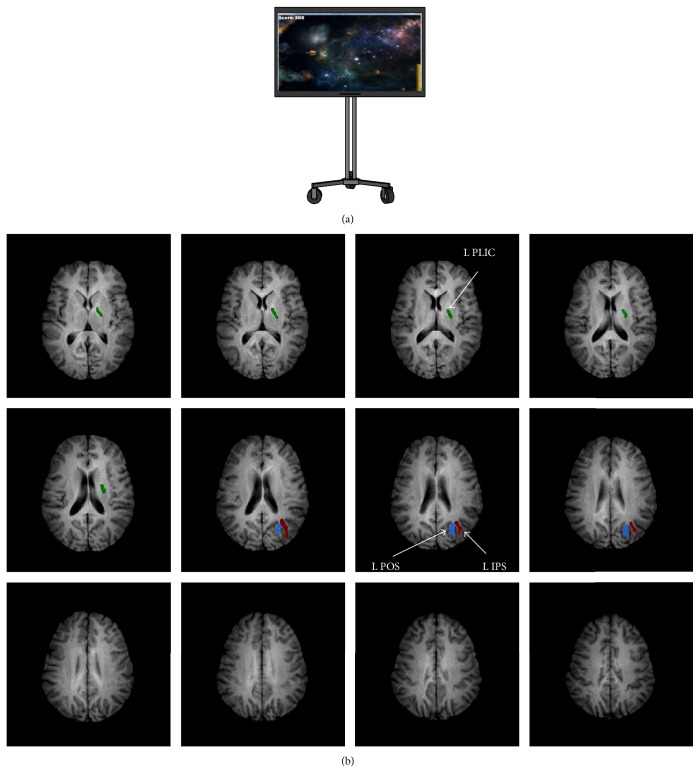
Experimental setup and region of interest locations. (a) Semi-immersive virtual reality game display with custom designed game software using the Microsoft Kinect sensor. (b) Representative example for voxel-based selection of each region of interest (displaying left regions only).

Sessions 2–11 involved motor practice of a semi-immersive virtual reality task that required skilled movements of the dominant right arm and integration of visual, spatial, and proprioceptive information. Dominant (right) arm use was manipulated through the performance of a semi-immersive virtual reality-based intercept and release task called TRAIT (Track and Intercept Task), which was performed in an interactive environment (outer space; Figure 1(a)). TRAIT employed a Microsoft Kinect sensor (Microsoft, Redmond, WA) using open source software, which tracks 3D joint movement. Arm motion during TRAIT was not constrained by the need to hold or manipulate a device. Participants were instructed to “save the world” by controlling an on-screen icon (spaceship) using movements of their dominant, right arm to intercept a moving object (an asteroid) as it emerged from the side of a 42 inches computer screen. Once intercepted, participants were required to accurately throw the object to hit a target (the sun). Participants progressed through 10 levels of increasing task difficulty as their skill improved.

Participants completed 10 TRAIT training sessions over a period of four weeks. During each session participants performed 5 blocks of the task. Each session lasted approximately 45 minutes. Each block contained 200 movements (100 object interceptions and 100 object throws). Participants completed 1000 skilled arm movements per session and a total of 10,000 experimental movements [14]. Task difficulty was manipulated by increasing the speed of the asteroid, decreasing the size of the asteroid or decreasing the size of the final target (the sun). The skill criterion to advance to the next level of task difficulty was an 80% success rate of object interception and hitting the target on two consecutive practice blocks. Levels of difficulty were designed to challenge a wide range of participants, maintain engagement, and prevent plateaus in performance.

Skill acquisition was quantified by exponentially fitting object interception time for each successful trial over the entire training period using (1) and extracting the rate of skill acquisition, overall change in movement time, and movement time at asymptote [15]:

(1) $$\begin{array}{c} E\left(\;{MT}_{N}\;\right)=A+Be^{-\alpha N}, \end{array}$$

where *E*(MT) is the expected value of movement time (MT) on trial *N*. *A* identifies the movement time at which the participant has plateaued in performance; *α* quantifies the rate of skill acquisition to the point of plateau and *B* is a measure of overall change in movement time from the beginning of training to the point of performance plateau.

### 2.3. MWF Analysis

T2 relaxation analysis used a nonnegative least-squares approach [16] with the extended phase graph algorithm [17] to partition the T2 signal using an unbiased approach based on the signal characteristics into short (15–40 ms), intermediate (40–200 ms), and long (>1500 ms) components using in-house software code (MATLAB R2010b, The MathWorks, Inc.) developed at the University of British Columbia. MWF was defined as the sum of the amplitudes within the short T2 component (15–40 ms) divided by the sum of the amplitudes for the total T2 distribution. Voxel-based maps were produced for each participant to evaluate MWF in the desired regions of interest (ROI). Tools in the FMRIB Software Library (FSL) [18] were used to generate the ROI masks. Initially, fractional anisotropy maps were generated using the dtifit component of FMRIB's diffusion toolbox [18]. These maps were then coregistered to the first echo of the T2 scan using an affine 12 parameter model in FLIRT [19] and then a two-timepoint percentage brain volume change was estimated with SIENA in a halfway space between the two images [18, 20]. The coregistered images were automatically segmented into gray matter, white matter, and cerebrospinal fluid using FAST [21]. The white matter segmentation mask was eroded by one voxel to remove any brain edge artifacts or subcortical gray matter tissue. Successful erosion was visually confirmed for each mask, which was subsequently used as the whole brain white matter (WBWM) mask.

Three a priori white matter regions of interest (ROIs) were selected for analysis bilaterally using a voxel-based approach for calculating MWF (Figure 1(b)) [22]. Regions of interest included the posterior limb of internal capsule (PLIC), the intraparietal sulcus (IPS), and the parieto-occipital sulcus (POS). A separate mask was generated for the segmented WBWM. These ROIs were selected based on the task characteristics and past work [2, 23, 24]. The PLIC contains the primary motor efferent fibers to the spinal cord for movement execution [25]. The IPS has been implicated in visually guided skilled movements and in attentional focusing during visuomotor integration [26]. The POS is involved in visuomotor coordination during arm reaching [27]. ROIs were manually delineated in FSLview using the coregistered fractional anisotropy map in the halfway space and then linearly registered to each MWF map [28]. The IPS was bound ventrally by the posterior thalamic radiation, dorsally by the angular gyrus, medially by the superior occipital gyrus white matter, and laterally by the lateral occipital gyrus. The POS was bound ventrally and medially by the cuneus, dorsally by the superior parietal lobule, and laterally by the occipital gyrus white matter bilaterally and the posterior limb of the internal capsule. Finally the PLIC was bound ventrally by the cerebral peduncle, dorsally by the splenium of the corpus callosum, laterally by the putamen and medially by the thalamus and thalamic nuclei, bilaterally. Two independent raters manually delineated each ROI and interrater reliability was assessed. Furthermore, to determine single-session test-retest reliability of MWF a subset of participants (*n* = 11) underwent a third MRI session, a minimum of three months following completion of the experimental protocol, in which two consecutive MCRI scans were performed using the same imaging sequence in order.

### 2.4. Statistical Analysis

All outcome measures met assumptions of normality and homogeneity of variance for parametric testing. First, motor skill acquisition was quantified with the three curve fitting variables: *A*, *B*, and *α*. Successful skill acquisition is represented with a positive *B* (change in movement time) value. Measures of skill acquisition were binned from training sessions 1–9 and training session 10 in order to identify potential relationships between early and late acquisition.

Second, single-sample *t*-tests were performed on the mean percent change values in MWF by ROI between pre- and postintervention timepoints. Next, planned bivariate correlation analyses were conducted between changes in MWF in the IPS and rate of skill acquisition. Statistical significance for all comparisons was set at *p* < 0.05.

## 3. Results

One participant was removed from data analyses due to a significant self-reported increase in physical activity concurrent with the training period which may have influenced overall myelin production [5].

In our control experiment, which measured the within-session test-retest reliability of the MWF signal, we noted that our MWF signal was stable as shown by excellent single-session test-retest reliability of MWF for each ROI (ICC range: 0.83–0.96; Table 1). Further, manual delineation of each ROI bilaterally also showed good to excellent interrater reliability between two raters (ICC range: 0.73–0.99).

### 3.1. Motor Skill Acquisition

**Figure 2:**
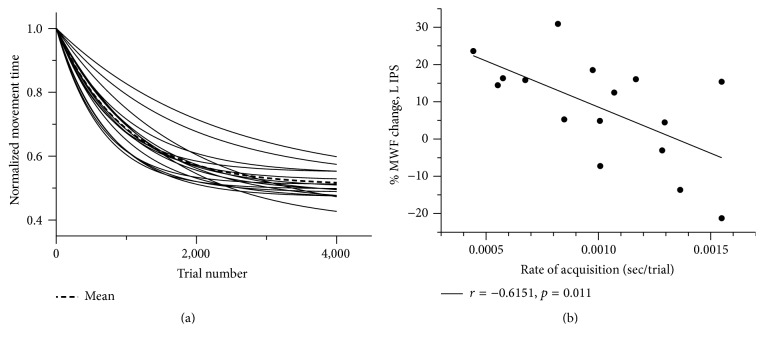
Relationships between MWF and skill acquisition. (a) Normalized acquisition curves for each individual participant and the group mean (dashed bold line) for successful trials. (b) Negative correlation between % MWF difference in the left IPS and rate of skill acquisition.

**Figure 3:**
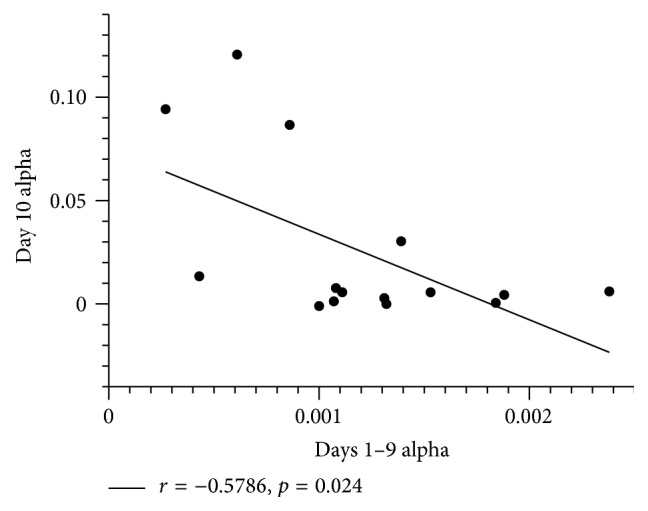
Early versus late skill acquisition. Negative relationship between rate of skill acquisition (alpha) during the first nine sessions of practice and final practice session.

Curve fitting revealed that all participants demonstrated acquisition of the motor task (positive *B* value; Figure 2(a)). Participants successfully intercepted an average of 81.3 ± 3.4% of the asteroids across all levels of task difficulty. There was a significant negative correlation between rate of skill acquisition during the first 9 sessions and the 10th session of training (*r* = −0.597, *p* = 0.019; Figure 3).

### 3.2. Myelin Water Fraction

**Figure 4:**
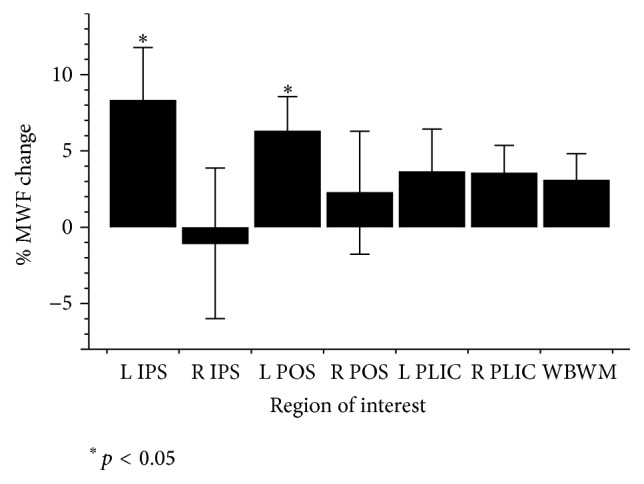
Changes in MWF by region of interest. Overall mean % MWF differences (±SEM) by region of interest and for WBWM.

The left IPS and left POS demonstrated significant MWF increases following training (IPS mean increase = 8.30% ± 13.90%; *t*_15_ = 2.39, *p* = 0.030 and POS mean increase = 6.28% ± 9.09%; *t*_15_ = 2.76, *p* = 0.014; Figure 4). MWF was not significantly increased in the other ROIs or within WBWM. A statistically significant negative correlation was observed between the percent difference in MWF in the left IPS and the rate of skill acquisition (*r* = −0.615, *p* = 0.011; Figure 2(b)).

## 4. Discussion

Our results revealed that increases in myelin were observed only in ROIs contralateral to the trained limb and these changes correlated with indices of skill acquisition over a four-week training period. Previous work exploring longitudinal changes in MWF has been performed in children during development [29] and in adults with multiple sclerosis [30]. However, the current data are the first to show changes in MWF following skilled motor practice that were associated with rate of skill acquisition.

Our study results extend previous findings from a rodent model of increased myelin in the contralateral motor cortex, following skilled upper-limb reaching, that were associated with rate of learning [4]. Recent work demonstrated that myelin plasticity following learning may increase conduction velocity and optimize timing in white matter circuitry [31]; it has been demonstrated that even a single session (~2 hours) of training can induce white matter plasticity [32]. Using a rodent model, McKenzie et al. (2014) discovered that oligodendrocyte generation is accelerated when learning a complex, new skill and that if oligodendrocyte precursor cells are genetically blocked, acquisition of the new skill remains incomplete [5]. Our data advance past work in humans that showed increased fractional anisotropy values in the IPS after complex bimanual skill (juggling) practice without finding relationship between fractional anisotropy values and skill acquisition [2].

In the current study, MWF in the left IPS correlated with indices of motor skill acquisition. It has been proposed that while acquiring new motor skills, there are discrete phases that must occur to facilitate memory consolidation and skill learning. The first and second phases tend to result in the largest changes in performance [33]. These early changes are followed by more gradual adaptations over subsequent training sessions [33, 34]. This progression reflects consolidation of the skill as it becomes more automated and requires less cognitive demand [33]. Our analysis model employed exponential curve fitting to characterize rates of change and allowed for the quantification of the entirety of performance during motor skill acquisition instead of relying on a simplified pre- to post-change score. We demonstrated that individuals with slower rates of skill acquisition in the first nine training sessions were faster to reach plateau on the 10th training session. Our findings suggest that individuals whose motor behaviour changed at a slower rate in the acquisition phase have the greatest rate of acquisition in later training sessions and importantly the greatest increases of MWF in the contralateral IPS. Given these relationships, we speculate that when initial task difficulty was high, the early acquisition phase was prolonged, promoting greater neuroplastic myelin changes in the left IPS.

Anatomically, the IPS is topographically organized and subregions of IPS demonstrate specific responses to discrete stimuli [35]. It has been demonstrated that the posterior IPS, in particular, is connected to extrastriate visual regions, which are controlled by visual attention [36]. Diffusion imaging demonstrated direct white matter anatomical projections from the IPS to the middle frontal gyrus [37] and previous work from our research group has implicated the middle frontal gyrus as part of a prefrontal network involved in sequence learning and motor control [38]. The middle frontal gyrus is a top-down processing region that is active during tasks with high attentional load [39]. As individuals gain mastery of a new movement, motor tasks becomes more automatic and activation in middle frontal gyrus is attenuated during task performance [40, 41]. Given the specific emphasis placed on visuomotor attention required to successfully complete the training task in the current study, specific parcellation of the posterior IPS may elaborate the specific loci of underlying myelin changes and should be a source for future investigation.

Our results suggest that myelin is modifiable by experience in humans. Increases in myelin may be aided by oligodendrogenesis associated with behavioral changes, as has been demonstrated in a mouse model using optogenetic stimulation [9]. Even modest increases in myelination may result in large increases in signal propagation speed resulting in more rapid information transfer between gray matter processing centers and improved synchrony between distant cortical regions [7]. These results provide insights into tissue-specific experience-dependent changes in white matter. Our findings may have important clinical implications and future investigations should evaluate the effect of intensive motor practice on myelin plasticity in individuals with neurologic conditions (e.g., stroke or those with demyelinating diseases such as multiple sclerosis) in order to assess the efficacy of behavioural training on myelin plasticity in the diseased brain.

## Figures and Tables

**Table 1:** Within-session (20 minutes apart) test-retest MWF reliability results (*n* = 11).

	Left			Right			WBWM
	IPS	POS	PLIC	IPS	POS	PLIC	—
Before MWF	0.041	0.124	0.169	0.059	0.153	0.172	0.106
After MWF	0.044	0.126	0.165	0.056	0.144	0.169	0.105
ICC	0.887	0.961	0.925	0.911	0.833	0.953	0.939
